# TTF-1/p63-Positive Poorly Differentiated NSCLC: A Histogenetic Hypothesis from the Basal Reserve Cell of the Terminal Respiratory Unit

**DOI:** 10.3390/diagnostics10010025

**Published:** 2020-01-06

**Authors:** Daniela Cabibi, Sandro Bellavia, Antonino Giulio Giannone, Nadia Barraco, Calogero Cipolla, Anna Martorana, Vito Rodolico, Massimo Cajozzo, Ada Maria Florena

**Affiliations:** 1 Department of Health Promotion, Mother and Child Care, Internal Medicine and Medical Specialties, Pathologic Anatomy Unit—University of Palermo, 90127 Palermo, Italy; cabibidaniela@virgilio.it (D.C.); bellaviasandro@yahoo.it (S.B.); anna.martorana@unipa.it (A.M.); vito.rodolico@unipa.it (V.R.); adamaria.florena@unipa.it (A.M.F.); 2 Department of Surgical, Oncological and Stomatological Disciplines—University of Palermo, 90127 Palermo, Italy; barraconadia@gmail.com (N.B.); calogero.cipolla@unipa.it (C.C.); massimo.cajozzo@unipa.it (M.C.)

**Keywords:** NSCLC, non-small-cell lung cancer, histogenetic hypothesis, TTF-1, p63, basal reserve cells, terminal respiratory unit

## Abstract

TTF-1 is expressed in the alveolar epithelium and in the basal cells of distal terminal bronchioles. It is considered the most sensitive and specific marker to define the adenocarcinoma arising from the terminal respiratory unit (TRU). TTF-1, CK7, CK5/6, p63 and p40 are useful for typifying the majority of non-small-cell lung cancers, with TTF and CK7 being typically expressed in adenocarcinomas and the latter three being expressed in squamous cell carcinoma. As tumors with coexpression of both TTF-1 and p63 in the same cells are rare, we describe different cases that coexpress them, suggesting a histogenetic hypothesis of their origin. We report 10 cases of poorly differentiated non-small-cell lung carcinoma (PD-NSCLC). Immunohistochemistry was performed by using TTF-1, p63, p40 (ΔNp63), CK5/6 and CK7. EGFR and BRAF gene mutational analysis was performed by using real-time PCR. All the cases showed coexpression of p63 and TTF-1. Six of them showing CK7+ and CK5/6− immunostaining were diagnosed as “TTF-1+ p63+ adenocarcinoma”. The other cases of PD-NSCLC, despite the positivity for CK5/6, were diagnosed as “adenocarcinoma, solid variant”, in keeping with the presence of TTF-1 expression and p40 negativity. A “wild type” genotype of EGFR was evidenced in all cases. TTF1 stained positively the alveolar epithelium and the basal reserve cells of TRU, with the latter also being positive for p63. The coexpression of p63 and TTF-1 could suggest the origin from the basal reserve cells of TRU and represent the capability to differentiate towards different histogenetic lines. More aggressive clinical and morphological features could characterize these “basal-type tumors” like those in the better known “basal-like” cancer of the breast.

## 1. Introduction

Identifying the correct type of lung cancer has become increasingly important due to the recent advances in “targeted” therapies. The morphological distinction between pulmonary adenocarcinoma (ADC) and squamous cell carcinomas (SCC) is sometimes difficult, mainly in cases of poorly differentiated tumors or when degenerative changes, necrosis and crushing may obscure the cell characteristics. Poorly differentiated non-small-cell lung carcinomas (PD-NSCLC) are tumors characterized by predominantly solid growth, formed by cells with large, eosinophilic cytoplasm, with a misleading tendency to keratinization or a pseudosquamoid morphology. Thus, histochemical detection of mucin and immunohistochemistry are often essential tests in differential diagnosis of NSCLCs, expanding the routine morphological examination. 

The combined use of antibodies TTF-1 and cytokeratin 7 (CK7) for ADC and p63 with high molecular weight keratins (CK5/6) for SCC provides a very reliable distinction between these two subtypes of non-small-cell carcinoma [1,2]. Recently, p40 has been considered the most specific marker of SCC [3]. SCC shows positive immunostaining for CK5/6, p63 and p40 (ΔNp63) (with strong and widespread nuclear reaction) and negative immunostaining for TTF-1 and CK7. In contrast, ADC shows positive immunostaining for TTF-1 and CK7 and negative immunostaining for p63, p40 and CK5/6 [3,4]. Even if few ADCs may stain positively for p63, they show only a focal and weak positivity [5], while CK5/6 and p40 expression is usually absent. CK5/6-positive immunostaining is very unusual in ADCs, and even if it is present, in the absence of p63 and p40, it is not sufficient to support the squamous differentiation [3,4,5,6]. For SCC, p63 and CK5/6, sensitivity varies from 75% to over 95% and their specificity varies from 70% to 100%. For ADC, TTF-1 is a highly specific marker (specificity 97–100%) but not very sensitive (sensitivity 54–75%), while CK7 is a more sensitive (usually >90%) but less specific marker (specificity 57–94%) [7,8]. It has also been reported that p40 has 100% sensitivity and specificity for squamous differentiation [9].

An immunohistochemical panel comprising TTF-1/p63 and, more recently, TTF1/p40 as first choice, has been considered sufficient for typifying the majority of non-small-cell cancers as ADC or SCC. Then, CK5/6 and CK7 could be added to the panel for cases with indeterminate results, which constitute only a small subset of cases [6,9]. Nevertheless, a small number of cases show the coexpression of some markers of both SCC and ADC differentiation. These cases are still poorly understood and their histogenesis is still not clear. In this study, we examined a small casuistry of PD-NSCLC coexpressing both TTF1 and p63, with the aims of better characterizing their immunohistochemical and genetic profile and suggesting a histogenetic hypothesis about their origin.

## 2. Materials and Methods

We describe 10 cases of poorly differentiated non-small-cell lung cancer (PD-NSCLC) retrieved from a sample of 230 NSCLC that were resected at the University of Palermo between 2014 and 2018. These cases constituted a small group in which coexpression of TTF1 and p63 had been found by immunohistochemistry previously performed during routine examination for diagnostic purpose and p63 and TTF1 coexpression was necessary for the cases to be included in the present study.

Hematoxylin and eosin (H&E)-stained TTF1 and p63 sections were retrieved from the archives of the Anatomic Pathology Unit of the University Hospital P. Giaccone of Palermo and were independently reviewed by two pathologists (DC and AGG). TTF1 and p63 immunohistochemical assays were repeated on new serial sections to confirm previous results, together with p40, CK5/6, CK7 and ALK immunostainings.

Immunohistochemistry was performed by a standard protocol on a BenchMark Ultra automated immunostainer according to the manufacturer’s instructions. Antigen retrieval was performed with CC1 buffer. Primary antibodies (Ventana-Roche^®^) included TTF-1 (anti-thyroid transcription factor-1 (clone 8G7G3/1)), p63 (clone 4A4), p40 (ΔNp63) (clone BC28), ALK (clone D5F3), CK5/6 (clone D5/16B4) and CK 7 (clone SP52). The slides were finally observed on Leica DM2000 optical microscope; microphotographs were obtained using a Leica DFC320 camera.

Genomic DNA (gDNA) was extracted from tumor specimens using QIA FFPE TISSUE KIT^®^, according to manufacturer’s instructions. Isolated gDNA was analyzed to evaluate DNA quantity using the NanoDrop^®^ Spectrophotometer.

To investigate the mutations of the EGFR, BRAF and all RAS genes, RT-PCR (TaqMan^®^ Mutation Detection Assay) was used. TaqMan^®^ Mutation Detection Assay is powered by castPCR™ technology, which refers to Competitive Allele-Specific TaqMan^®^ PCR. The CastPCR™ technology is highly specific and sensitive, able to detect rare amounts of mutated DNA in a sample containing large amounts of normal, wild-type gDNA.

The study was conducted in accordance with the Declaration of Helsinki. Further ethical statement is not applicable because this was a retrospective study with immunohistochemical staining on absolutely anonymous data.

## 3. Results

### 3.1. Clinical Data

All the patients were men and were strong smokers (age range between 54 and 80 years).

In each of them, chest X-ray and computed tomography (CT), with and without contrast, showed a peripheral radiopaque lesion, with irregular margins with diameter ranging from 1.9 to 6.2 centimeters. In all patients, bronchoscopy showed no abnormalities of the bronchial mucosa and the transbronchial biopsies were negative for tumor, while the CT-guided transthoracic biopsy evidenced the presence of a poorly differentiated carcinoma in all cases. Neoadjuvant radiotherapy and/or chemotherapy were not administered before surgery. 

### 3.2. Histological Data

**Figure 1:**
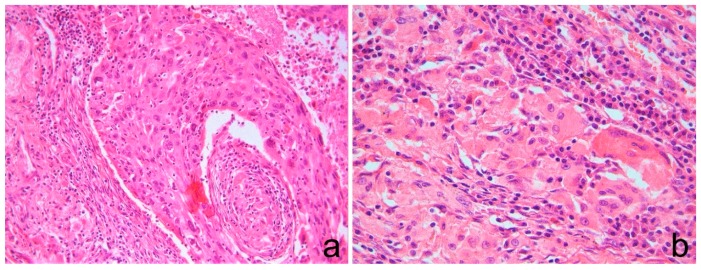
(**a**) Neoplastic solid nests formed by (**b**) cells with large, eosinophilic cytoplasm with an apparent tendency to keratinization. (**a**,**b**) Hematoxylin and eosin stain. Original magnification: (**a**) 200×; (**b**) 400×.

All the cases consisted of PD-NSCLC showing solid nests formed by cells with a large, eosinophilic cytoplasm, with apparent tendency to keratinization (Figure 1). No mucus secretion was evidenced.

In all the cases, the morphology indicated a PD-NSCLC more suggestive of a squamous histotype.

The immunohistochemical examination, performed on serial sections, confirmed in all the cases coexpression of p63 and TTF-1 in more than 60% of the cellular elements. Noteworthily, both antibodies were present in the nuclei of the same cellular elements.

**Figure 2:**
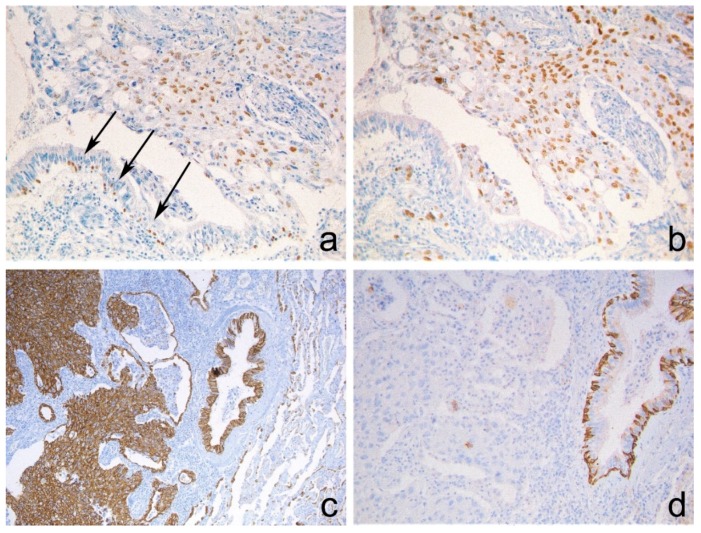
Positive immunostaining for (**a**) p63, (**b**) TTF-1 and (**c**) CK7 and negative immunostaining for (**d**) CK5/6 in neoplastic infiltrating areas. p63 and CK5/6 stained positively the basal layer of the terminal bronchiolar epithelium (**a**, arrows; **d**, right side). (**a**–**d**) Immunoperoxidase stain. Original magnification: (**a**,**b**,**d**) 200×; (**c**) 100×.

Six cases also showed strong and widespread positive immunostaining for CK7 and negativity for CK5/6 (Figure 2).

**Figure 3:**
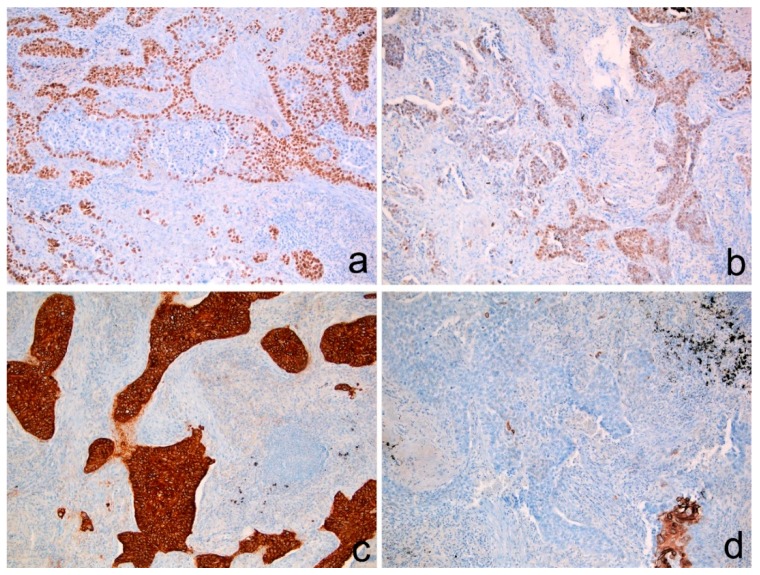
Positive immunostaining for (**a**) p63, (**b**) TTF-1 and (**c**) CK5/6 in neoplastic infiltrating areas. (**d**) CK7 was absent in most areas. (**a**–**d**) Immunoperoxidase stain. Original magnification: (**a**–**d**) 100×.

The other ones showed strong and diffused positivity for CK5/6, whereas CK7 was only focally expressed. P40 stained negatively in all cases. (Figure 3).

**Figure 4:**
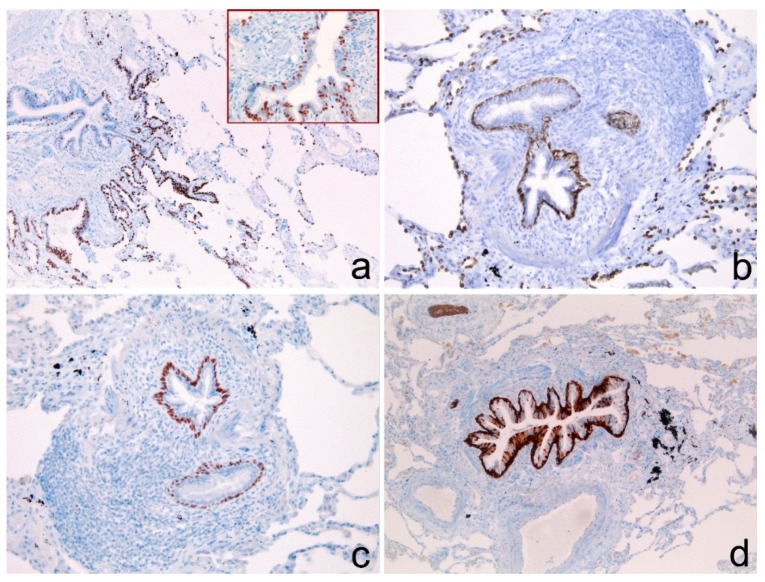
(**a**,**b**) Basal reserve cells of the terminal bronchioles positive for TTF-1. The insert shows TTF1-positive basal cells at higher magnification. Note TTF1-positive immunostaining also for the pneumocytes II (insert). Serial sections stained for (**c**) p63 and (**d**) CK5/6 showed positive immunostaining in basal reserve cells of TRU. (**a**–**d**) Immunoperoxidase stain. Original magnification: (**a**–**d**) 100×; (**b**) 200×; insert: 400×.

It is worth noting that, in all cases, the basal cells of the epithelium of the distal terminal bronchioles stained positively for TTF-1, p63 and CK5/6 (Figure 4a–d). TTF1 stained positively also the alveolar epithelium that could be used as internal positive control. (Figure 4).

In accordance with the immunohistochemical findings, all these cases were defined as solid variant adenocarcinomas with unusual coexpression of TTF-1 and p63. Moreover, in 4 out of 10 cases, a tendency towards squamous differentiation was evidenced by the unusual coexpression of CK5/6 together with TTF-1 and p63.

The real-time PCR performed for EGFR and BRAF gene mutational analysis showed a wild-type genotype in all cases. Two of our cases were KRAS mutated.

## 4. Discussion

In lung ADCs, positive immunostaining for p63 is rare; when present, it has been reported as weak and focal [5,9,10,11,12,13]. Only in 5.5% of cases has coexpression of both TTF-1 and p63 in the same cellular elements been reported [10,11,12,14,15].

In adenosquamous carcinomas both p63 and TTF-1 can be present, but they have been evidenced in different areas of the tumor. The morphological aspect of our cases was more suggestive of squamous differentiation, but the immunohistochemical assay showed nuclear coexpression of p63 and TTF-1 in the same cells, with p63 positivity in more than 60% of elements. The p63-positive immunostaining was strong and diffuse and not weak and focal, as reported in the above-cited studies.

Moreover, in 6 out of 10 cases the neoplastic cells were positive for CK7 and negative for CK5/6. The presence of TTF-1 and cytokeratin 7, the latter considered a marker of glandular differentiation, [16] along with the negativity for CK5/6 and p40, led us to classify these case as “ADC, solid variant”, despite the morphological aspects that could suggest SCC. On the other hand, the strong and widespread positivity for CK5/6 in 4/10 cases suggests a tendency towards squamous differentiation, even if these cases must be defined ADC, in keeping with the presence of TTF1 expression and p40-negative immunostaining.

In non-neoplastic tissue, p63 is expressed in the basal reserve cells of stratified squamous epithelia and of gland epithelium [11,12]. The positivity of p63 has even been reported in the terminal bronchioles [13]. Instead, TTF-1 is diffusely expressed both in the alveolar epithelium and in the basal cells of the epithelium of the distal bronchioles, which constitute the terminal respiratory unit (TRU) [17]. So, TTF-1 is considered the most sensitive and specific marker to define ADC originating from the TRU, usually arising in female non-smokers and often harboring EGFR mutations [18].

In 2002, Yatabe et al. [19] stated that “adenocarcinomas with TRU morphology, consisting of elements similar to the pneumocytes II order, were positive for TTF-1 in 88% of cases”, in contrast with adenocarcinomas without TRU morphology. On this basis, Maeshima et al. suggested that TTF-1-positive tumors derive from the peripheral alveolar epithelium. They acquire heterogeneity and undergo differentiation during development, towards a phenotype that can be similar to that of Clara cell/pneumocytes II type, mixed (Clara cell/bronchial), superficial bronchial-epithelium-like or poorly differentiated, but always expressing TTF-1. As 80% of poorly differentiated carcinomas are positive for TTF-1, the authors suggest that the majority of them may derive from the peripheral alveolar epithelium of the TRU [20,21]. 

TRU has been defined as composed of alveolar cells and nonciliated epithelium of the distal bronchioles. To our knowledge, no attention has yet been given to the presence of the basal reserve cells of TRU and to a hypothetic role of them in cancer histogenesis.

In keeping with Yatabe et al. [19] and with Maeshima et al. [20] we hypothesized that cases of PD-NSCLC showing TTF-1 positivity could originate from TRU. Nevertheless, because of the coexpression of TTF-1 and p63 and the lack of EGFR mutations, we judge an origin from the superficial epithelial cells of the alveolar and bronchiolar TRU lining to be unlikely. We hypothesize an origin from the basal reserve cells of the TRU that show positive immunostaining for TTF1 and p63, overlapping with the results obtained in our 10 cases of PD-NSCLC. This hypothesis has been previously suggested by Wu et al. [12], limited to previously called “brochiolo-alveolar carcinomas” and “well-differentiated adenocarcinomas (WD-ADC)”, but not for PD-NSCLC. Moreover, TTF-1 was not assessed in their study. Noteworthily, in four of our cases, the positive immunostaining of neoplastic cells for CK5/6 and p63, but not for CK7, could indicate the persistence of the some of the markers typical of the multipotent basal reserve cells of TRU or a potential divergent early squamous differentiation.

Recently, two cases of NSCLCs with widespread and strong nuclear positivity for TTF-1 and p40 have been reported [21,22]. The authors speculate that these cases “could result from expression of stem/progenitor cell plasticity” [21]. Pelosi et al. proposed the term “adenosquamous carcinoma or at least NSCLC with adenosquamous immunophenotype” for these cases [21], while Hayashi et al. suggested that they could represent a “new under-recognized entity” [22].

In our study we observed that the basal reserve cells of TRU are p40-positive as well. We hypothesize that very rare TTF1/p40-positive PD-NSCLC could represent cases in which the immunophenotypical features of the basal reserve cells are still maintained.

This last hypothesis, if confirmed, could support the histogenetic linkage with TRU basal reserve cells of this “under-recognized entity”. In fact, to our knowledge, no lung structures show a TTF1/p63/p40-positive immunophenotype other than basal reserve cells of TRU. However, TTF1/p40-positive PD-NSCLC cases are much rarer than TTF1/p63-positive PD-NSCLC cases and were not present in our sample.

## 5. Conclusions

**Figure 5:**
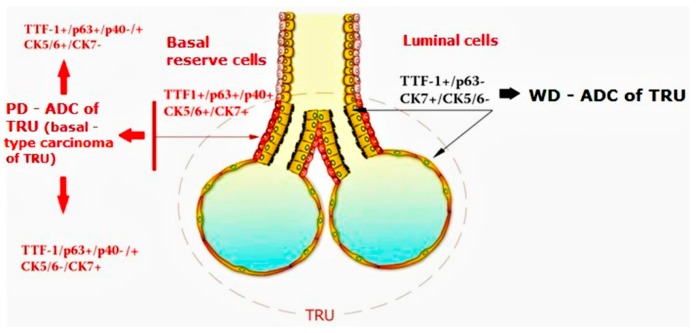
On the right, the scheme illustrates the origin of well-differentiated adenocarcinoma (WD-ADC) from the superficial epithelial cells of the alveolar and bronchiolar lining of the terminal respiratory unit (TRU) (in yellow). On the left is shown the hypothesis about the origin of solid, TTF1+/p63+/p40+/− poorly differentiated adenocarcinoma (PD-ADC) from the basal reserve cells of the TRU (in red), coexpressing p63, TTF-1 and, in rarer cases, p40.

To our knowledge, this is the first study in which p40 expression has been evidenced in the basal reserve cells of TRU and the first one in which the coexpression of p63 and TTF1 in PD-NSCLC has been related to the origin from the reserve basal cells of the TRU. We agree with Hayashi et al. about the hypothesis that TTF1+/p63+/p40−/+ cases could represent “a new, under-recognized entity” and we propose to name them “basal-type TRU carcinoma” (Figure 5). In our cases, p40 was always negative, perhaps because the differentiation towards ADC was more pronounced, as suggested by the immunophenotype CK7+/CK5/6-negative in 6 out of 10 cases. Otherwise, the hypothesis that the differentiation towards squamous phenotype arrested in a very early stage could be suggested. All 10 patients had an aggressive clinical course and all developed metastases within 1 year from the first diagnosis, with an overall survival less than 5 years. All of them were wild-type for EGFR, ALK and BRAF. Two of them were KRAS mutated. So, in clinical features and molecular gene profile, they differ from the TRU-b-type adenocarcinomas previously described, that have been reported to be TTF1 positive [23].

In keeping with TTF1 expression, we hypothesize that these tumors arise from the TRU and share some features of TRU-a-type tumors described by Takeuchi [23] and other ones of non-TRU-type tumors, such as the aggressive clinical and morphological features, the absence of EGFR, ALK and BRAF mutation, the occasional presence of KRAS mutation. Finally, both the aggressive behaviors and their hypothetic origin from the basal cells could be similar to those of the better known “basal-like” breast cancer. This cancer arises from the basal reserve cells of terminal ductular lobular unit (TDLU) and is defined by expression of high weight cytokeratins (CK5/6, CK14, CK17) and epidermal growth factor receptor (*EGFR*) and by the lack of expression of the hormone receptors and of human epidermal growth factor receptor 2 (*HER2*). “Basal-like” breast cancer has an aggressive behavior and poor prognosis [24,25,26,27,28].

The study has some limits, with the main limit being the low number of cases, due to the rarity of this group of neoplasias. Additional multicentric studies on larger series with the aid of new technologies (i.e., next generation sequencing (NGS)) could confirm clinicopathological and genetic features of these rare tumors along with their histogenetic linkage with the basal cells of TRU in order to verify that they constitute a true subtype of lung adenocarcinomas.
